# Identification of the Wallenda JNKKK as an Alk suppressor reveals increased competitiveness of Alk-expressing cells

**DOI:** 10.1038/s41598-020-70890-6

**Published:** 2020-09-11

**Authors:** Georg Wolfstetter, Kathrin Pfeifer, Mattias Backman, Tafheem A. Masudi, Patricia Mendoza-García, Sa Chen, Hannah Sonnenberg, Sanjay K. Sukumar, Ezgi Uçkun, Gaurav K. Varshney, Anne Uv, Ruth H. Palmer

**Affiliations:** 1 Department of Medical Biochemistry and Cell Biology, Institute of Biomedicine at the Sahlgrenska Academy, University of Gothenburg, 41390 Gothenburg, Sweden; 2 Department of Molecular Biology, Umeå University, 90187 Umeå, Sweden; 3 Genes & Human Disease Program, Oklahoma Medical Research Foundation, Oklahoma, OK 73104 USA

**Keywords:** Non-small-cell lung cancer, Cancer microenvironment, Cancer models, Oncogenes, Cell death, Cell signalling, Differentiation, Genetic interaction, Mutation, Transgenic organisms, Drosophila, Genetic mapping

## Abstract

Anaplastic lymphoma kinase (Alk) is a receptor tyrosine kinase of the insulin receptor super-family that functions as oncogenic driver in a range of human cancers such as neuroblastoma. In order to investigate mechanisms underlying Alk oncogenic signaling, we conducted a genetic suppressor screen in *Drosophila melanogaster*. Our screen identified multiple loci important for Alk signaling, including members of Ras/Raf/ERK-, Pi3K-, and STAT-pathways as well as *tailless (tll)* and *foxo* whose orthologues NR2E1/TLX and FOXO3 are transcription factors implicated in human neuroblastoma. Many of the identified suppressors were also able to modulate signaling output from activated oncogenic variants of human ALK, suggesting that our screen identified targets likely relevant in a wide range of contexts. Interestingly, two misexpression alleles of *wallenda* (*wnd,* encoding a leucine zipper bearing kinase similar to human DLK and LZK) were among the strongest suppressors. We show that Alk expression leads to a growth advantage and induces cell death in surrounding cells. Our results suggest that Alk activity conveys a competitive advantage to cells, which can be reversed by over-expression of the JNK kinase kinase Wnd.

## Introduction

The receptor tyrosine kinase anaplastic lymphoma kinase (ALK) was initially described as oncogenic driver in anaplastic large-cell lymphoma and is implicated in a wide range of cancer types including non-small cell lung cancer and neuroblastoma^1^. During development, the vertebrate ALK receptor is activated by ALKAL (also known as FAM150 or Augmentor) family proteins while LDL-domain protein ligands activate Alk in invertebrates^2–7^. In contrast, cytosolic ALK fusion proteins, such as NPM1-ALK and EML4-ALK, are constitutively active and common drivers of tumor progression in anaplastic large-cell lymphoma and non-small cell lung cancer^1,8–10^. These ALK fusion proteins are ligand-independent and often ectopically expressed in tissues that would normally not express ALK^1^. In addition to the activation of ALK by fusion events, the full length receptor can be activated by point mutations in its kinase domain which has been described in familial and sporadic neuroblastoma^1,11–16^. Clinical studies suggest that ALK activation drives tumor growth and indeed ALK-driven cancers respond well to initial therapy with ALK tyrosine kinase inhibitors^17^. However, ALK-driven tumors frequently become resistant to ALK inhibitor treatment, underscoring a need to better understand signaling events that occur during aberrant ALK activity.

To shed light on these signaling events, we and others have used various model organisms, such as *Drosophila melanogaster*, to complement in vitro cell culture studies. In *Drosophila,* a single Alk orthologue is required to specify the muscle founder cells of the larval gut musculature^2,3,18^. In addition, Alk signaling plays a complex role in many aspects of neuronal function^19–25^. Interestingly, ectopic expression of Alk in the developing fly eye results in a characteristic phenotype reflecting the activation state of the receptor^26,27^. Therefore, the fly eye has become a valuable model system to analyze putative activating human ALK mutations identified in neuroblastoma patients^28–31^. The sensitivity of the fly eye model to both *Drosophila* and human ALK activity suggests that a genetic modifier screen in the fly holds the potential to reveal important downstream factors of Alk signaling in vivo. We crossed flies ectopically expressing Alk under control of the *sevEP-Gal4* driver to flies carrying genetically mapped deficiencies from the DrosDel collection^32^ and scored progeny for suppression or modification of the Alk-induced eye phenotype. Among the strongest suppressors, we identified gain of function alleles of the *LZK, MAP3K13* (*leucine zipper bearing kinase, mitogen-activated protein kinase kinase kinase 13*) and *DLK, MAP3K12* (*dual leucine zipper kinase, mitogen-activated protein kinase kinase kinase 12*) homolog Wallenda (*wnd*). Wnd has previously been shown to regulate JNK-mediated cell death in *Drosophila*^33^. JNK signaling in *Drosophila* tumorigenesis models can promote either cell-invasion or apoptosis dependent on context^34,35^. Investigating cell death in the context of Alk expression revealed increased cell death in Alk-negative neighboring cells, suggesting that ectopic Alk signaling leads to a competitive advantage that is further strengthened by the finding that Alk-expressing clones have a slight, but significant, growth advantage. This Alk-dependent increased competitiveness can be abrogated by elevated levels of JNK activation, through expression of Wnd or the JNKK Hemipterous (*hep*) in Alk expressing cells, suggesting that Wnd restrains Alk anti-apoptotic signaling output via modulation of JNK.

## Results

### Ectopic expression of Alk interferes with Drosophila eye development

**Figure 1:**
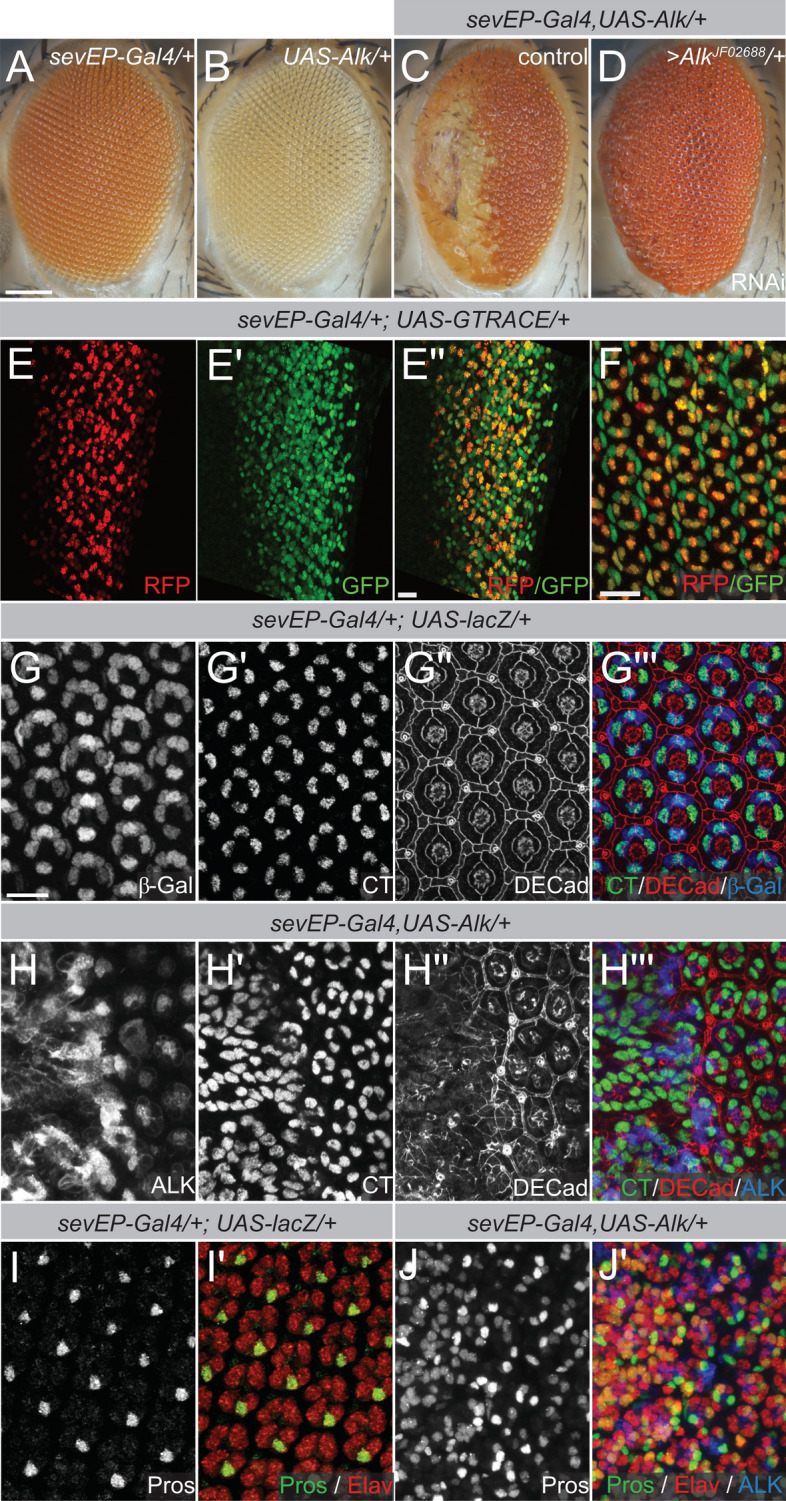
Ectopic expression of *UAS-Alk/* + with the *sevEP-Gal4* driver interferes with normal eye development. (**A**–**D**) Eyes of adult female flies with the indicated genotypes are shown. n > 200, 100% penetrant (**A**) “Wild-type” eye morphology of flies with either one copy of the *sevEP-Gal4* driver insertion or (**B**) one copy of the *UAS-Alk* transgene. (**C**) Ommatidial disruption and necrotic scars in the anterior eye upon *sevEP-Gal4/* + induced *UAS-Alk/* + expression. The posterior half exhibits fused ommatidia and missing bristles. (**D**) Phenotypic rescue by co-expression of an Alk-specific RNAi. (**E**,**F**) G-TRACE analysis. Real-time *sevEP-Gal4* expression is indicated by RFP (red) while continued expression in the lineage is indicated with GFP (green). (**E**–**E’’**) G-TRACE analysis in larval eye discs and in pupal eye discs (**F**). (**G**–**J’**) Immunofluorescence staining of eye discs from pupae (50 h apf) of the indicated genotypes. Antibody staining against Cut (CT) and *Drosophila* epithelial cadherin (DECad) reveals increased numbers of CT-positive cells and loss of ommatidia organization upon ectopic *Alk* expression (quantified in Fig. S1). (**G**,**G’’’**) βGalactosidase (βGal) reveals driver activity in a control eye disc, (**H**,**H’’’**) anti-ALK staining reveals transgene expression. Elav labels all photoreceptor cells and anti-Prospero (Pros) staining was used to identify R7 cells (**I**–**J**’). (**J**,**J**’) Upon ectopic expression of *UAS-Alk* the number of R7 photoreceptors is increased (quantified in Fig. S1). Scale bars are 100 µm in (**A**) and 10 µm in (**E’’**) and (**F**,**G**); anterior is left in all images.

We previously showed that the signaling activity of *Drosophila* and human ALK receptors can be assessed in the fly eye^4,26–31,36^. When *Drosophila* Alk was ectopically expressed in the developing eye under control of the *sevEP-Gal4* driver, we observed severe loss of ommatidia and patches of necrotic tissue in the anterior part of the adult fly eye as well as a “rough-eye–morphology” in the posterior part (Fig. 1A–C). This phenotype was suppressed when co-expressing an RNAi construct (*UAS-Alk*^*JF02688*^) targeting Alk (Fig. 1D). In order to characterize the *sevEP-Gal4* driver further, we performed lineage analysis using the G-TRACE system^37^, which revealed driver activity posterior to the morphogenetic furrow in third instar larval eye discs (Fig. 1E–E’’). The presence of *sevEP-Gal4* > *UAS-RFP*-expressing and EGFP-positive, lineage derived cells suggested that the *sevEP* reporter resembles the transient expression pattern of *sevenless* in early photoreceptors and remains active only in a subset of retinal cells^38–40^. This observation was more apparent at later pupal stages when G-TRACE analysis indicated continued RFP expression in the R7 photoreceptor and the cone cells that are recruited later to the ommatidial cluster while other cells of the ommatidium solely expressed EGFP (Fig. 1F). To further analyze the effects of ectopic Alk expression on eye development, we dissected eye discs 50 h after pupa formation (apf) (Fig. 1G–J’). Activity of the *sevEP-Gal4* driver revealed by *UAS-lacZ* (Fig. 1G,G’’’) resembled the previously described pattern of *sevenless* in R1, R6, R7 photoreceptors and cone cells^38–40^. In control eye discs, DECad staining indicated a regular arrangement of ommatidia upon β-Galactosidase expression (Fig. 1G’’,G’’’). Cut (CT), a lineage marker for cone cells, was expressed in four cells of each ommatidium (Fig. 1G’,G’’’; quantified in Fig. S1A,B). The neuronal marker Elav was expressed in all photoreceptor cells (Fig. 1I’) and Pros was expressed only in R7 cells (Fig. 1I,I’; quantified in Fig. S1C,D). In *sevEP-Gal4* > *UAS-Alk/* + eye discs, the expression pattern of these markers was highly irregular (Fig. 1H–H’’’,J–J’). A high number of Alk-expressing cells in the anterior region (Fig. 1H,H’’’) correlated with a high degree of ommatidia disorganization as revealed by DECad staining (Fig. 1H’’,H’’’) while, in the posterior region, ommatidia could be identified but were partially fused and irregular (Fig. 1H’’,H’’’). Moreover, the numbers of CT- and Pros-positive cells were significantly higher in *sevEP-Gal4* > *UAS-Alk/* + eye discs than in controls (Fig. 1G’,H’,I,J; quantified in Fig. S1A–D). This increase was most pronounced in anterior regions of the eye discs (Fig. 1H’,J; quantified in Fig. S1A–D). Taken together, this analysis suggests that ectopic expression of Alk leads to aberrant pattern of expression of lineage markers and interferes with ommatidia differentiation in the developing eye disc.

### A genetic modifier screen identifies targets of Alk-signaling in Drosophila

**Figure 2:**
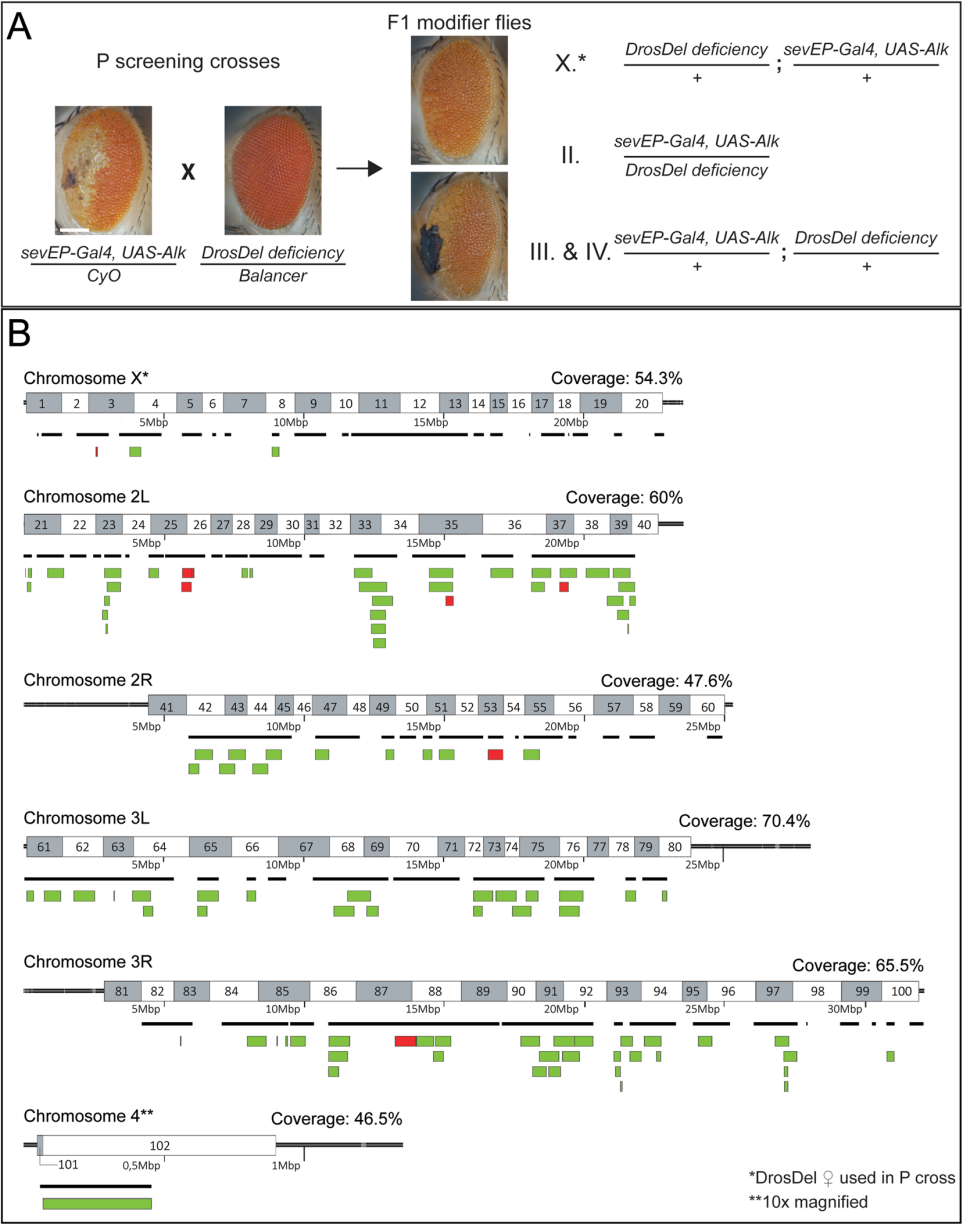
A deficiency screen identifies modifiers of ectopic Alk signaling. (**A**) Layout of the deficiency screen to identify modifiers of the *sevEP* > *, UAS-Alk/* + induced eye phenotype. Scale bar = 100 µm. (**B**) Schematic representation of chromosome maps including coverage by the DrosDel deficiencies used in the screen (black lines). Suppressor regions appear *green*; deficiencies that modified the phenotype otherwise are depicted in *red*.

We conducted a genetic modifier screen (Fig. 2A) based on the rationale that dose-reduction of factors acting downstream of Alk would modify the observed eye phenotype. We first tested a number of potential suppressors in a validation screen (Fig. S2). These included loss-of-function (lof) alleles of *jelly belly* (*jeb*) encoding the Alk-activating ligand in *Drosophila*^2,3,18,41^, and RTK-associated signaling molecules such as *C3G*^42,43^, *Son of sevenless* (*Sos*)^44^, *14-3-3ε* ^45^, *Ras oncogene at 85D* (*Ras85D*)^46^, *kinase suppressor of Ras* (*ksr*)^47^, *rolled* (*rl*) encoding for *Drosophila* ERK1,2^48^, and *pointed* (*pnt*)^49^. When crossed into the *sevEP-Gal4* > *UAS-Alk/* + background, single copies of these alleles suppressed the eye phenotype to varying degrees (Fig. S2A-H), indicating that genome-wide screening of deficiency strains should allow us to identify genomic loci implicated in Alk signaling.

**Figure 3:**
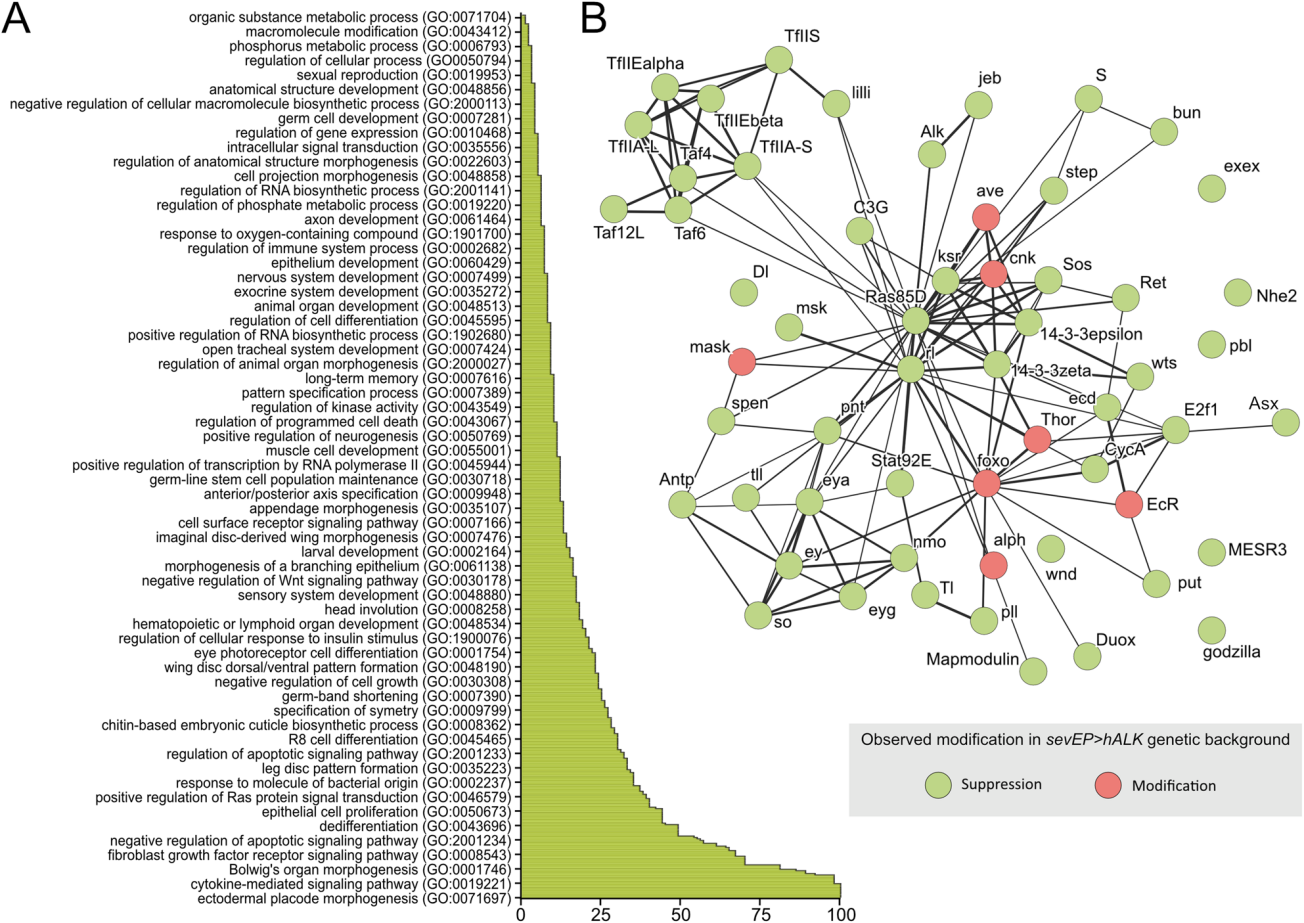
Candidate genes revealed by the modifier screen. (**A**) Graphic representation of a PANTHER4.0-driven gene ontology (GO) enrichment analysis of Alk modifying loci. (**B**) String analysis of the Alk modifier network. For visualization purposes, the line thickness represents the strength of support for an interaction within the network. Suppressors are shown in *green*; other modifiers are depicted in *red*. Details of the individual alleles tested are found in Table S1B.

We screened 388 lines from the DrosDel collection of deficiencies^50^ covering about 60% of the annotated *Drosophila* genome and scored 58 modifying genomic regions covered by 133 deficiencies (Fig. 2B, green; Table S1). Ninety-eight deficiencies suppressed the Alk-induced eye phenotype while ten deficiencies led to other modifications such as enhanced necrosis in the eye (Fig. 2B, red; Table S1). We did not obtain adult progeny with 25 deficiencies, of which most were located on the X-chromosome. Further genomic mapping revealed 38 individual loci within these deficiencies that modified the Alk-induced eye phenotype (Fig. 3B, Table S1, see also discussion section). Based on this information, we further tested related factors leading to the identification of 12 additional loci that were not covered by the originally screened DrosDel deficiencies (Table S1). For several modifying deficiencies we were not able to map down individual loci that modified the Alk-induced eye phenotype (Table S1). PANTHER14.0-driven gene ontology (GO) enrichment analysis (https://geneontology.org/, Dec 2019)^51^ of all Alk modifiers (Fig. 3) revealed a high abundance of factors associated with optic placode development and signal transduction as well as cell dedifferentiation and negative regulation of apoptosis and autophagy (Fig. 3A, Table S1). Among these modifiers, we identified Ras/Raf/ERK network components such as *MESR3*, *cnk* and *aveugle* (*ave,* also referred to as *hyphen*) (Fig. 3B). Other identified signaling molecules included *PI3K21B*, *STAT92E*, the protein serine/threonine phosphatase *alphabet* (*alph*), *split ends* (*spen*), *Star* and *moleskin* (*msk*) (Fig. 3B). A number of transcription factors also modified the Alk-induced eye phenotype including the forkhead box, sub-group O transcription factor *foxo*, *tailless* (*tll*), *eyeless* (*eye*) and *eyes absent* (*eya*). We also identified *lilliputian* (*lilli*) and factors involved in regulation of RNA polymerase II-induced transcription (*TFIIA-S*, *TFIIA-L*, *TFIIEα*, *TFIIEβ* and the TFIID-associated factors *Taf4*, *Taf6*, *TFIIS*, *Taf12L*) (Fig. 3B).

In some cases, more than one gene within a suppressing deficiency was found to suppress Alk-mediated signaling. For example, while mapping down the suppressor contained within deficiency *ED6346* we noticed that mutations in both the *tailless (tll)* transcription factor^52^ and the *warts/lats* serine/threonine kinase^53,54^ suppressed Alk. We also noticed that certain balancer chromosomes modified the *Alk*-induced phenotype. In case of the *CyO* chromosome, we identified mutations in the *Duox* (*Curly*) gene as moderate suppressors (Table S1).

### The activity of human oncogenic ALK is modulated by the identified modifiers

**Figure 4:**
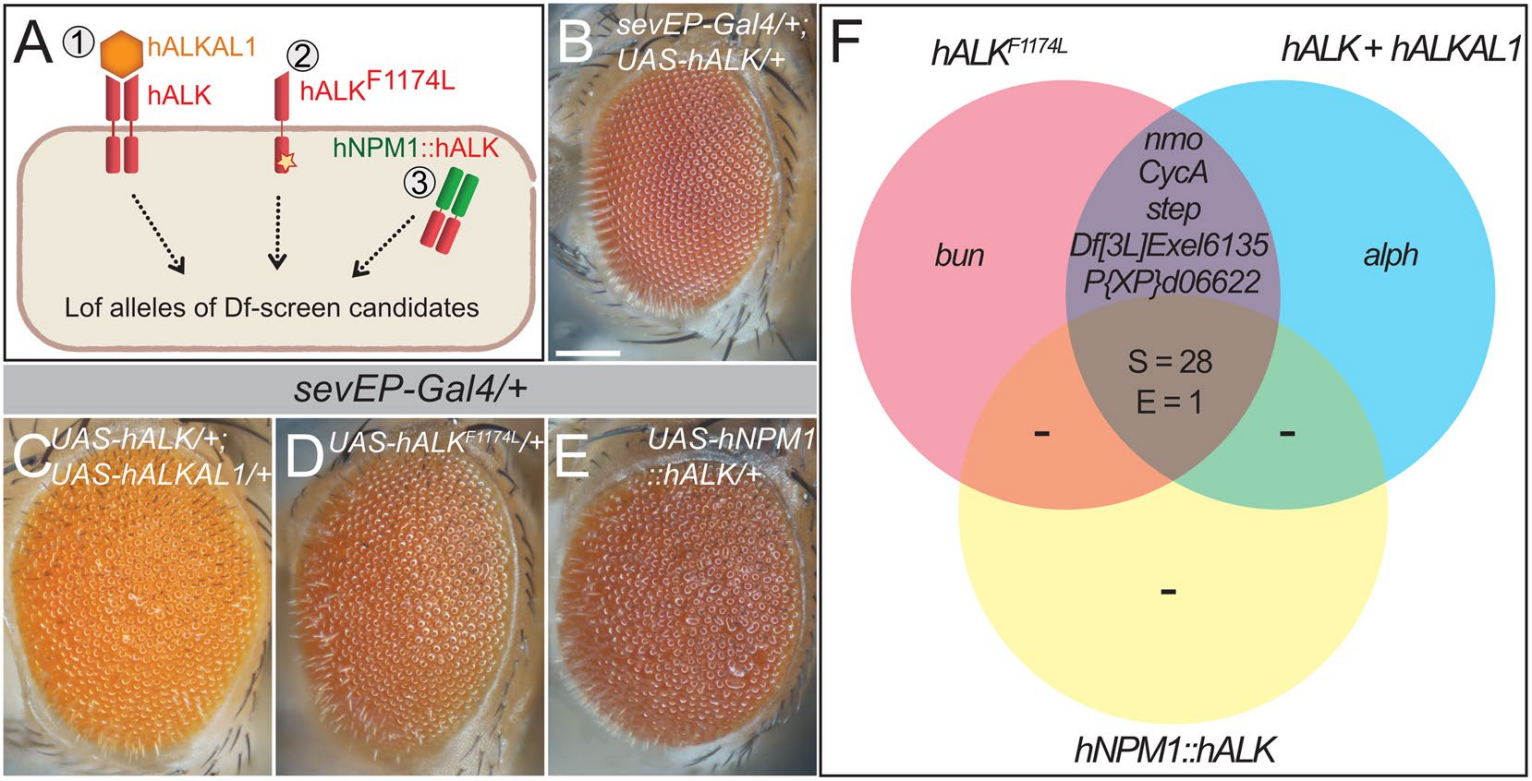
Human *ALK* variants are suppressed by mutations in the identified candidate genes. (**A**) Schematic representation of the screening approach, testing (1) hALK with the ALKAL1 ligand, which leads to high levels of ALK signaling activity, (2) a constitutively active hALK-F1174L observed in neuroblastoma and (3) the NPM1::ALK oncogene found in anaplastic large cell lymphoma. Single copies of loss-of-function (lof) mutations in the identified candidate genes were tested for phenotypic modification in flies ectopically expressing one of three oncogenic variants of human ALK (hALK) under *sevEP-Gal4* control. (**B**–**E**) Eyes of adult female flies expressing either wild-type hALK (**B**), wild-type hALK activated by ALKAL1 (**C**), the hALKF1174L activating point mutation (**D**) or the chimeric hNPM1::hALK protein (**E**) with *sevEP-Gal4*. Scale bar = 100 µm. (**F**) Venn diagram representation of the results of the hALK modifier screens: 28 alleles suppressed (S) the rough-eye phenotype of all hALK variants (corresponding to the *14-3-3-ε, 14-3-3ζ, Asx, Cul2, Dl, Duox, E2f1, eya, eyg, ksr, lilli, Mapmodulin, msk, pll, pnt, Ras85D, rolled, Sos, Star, Stat92E, Taf6, TfIIA-L, TFIIA-S, TFIIEa, tll,* and *wts* loci). One allele affecting two of the predicted five *EcR* isoforms enhanced (E) the eye phenotype. Modifiers that did not affect all hALK variants are highlighted in the diagram.

Aberrant ALK activation occurs in a range of cancers^55^. We therefore tested whether the modifiers found in our screen were also able to modulate signaling output from human ALK. To do this we activated human ALK (Fig. 4A) in three ways by ectopically expressing: (1) human ALK with the ALKAL1 ligand, which leads to high levels of ALK signaling activity^4^ (Fig. 4A,C), (2) a constitutively active ALK variant (ALK-F1174L) commonly observed in neuroblastoma patients^12–16^ (Fig. 4A,D), and (3) the chimeric NPM1::ALK oncogene found in anaplastic large cell lymphoma^8^ (Fig. 4A,E). When expressed in the fly eye under control of the *sevEP-Gal4* driver, we observed a rough eye phenotype in all three scenarios but not the severe loss of ommatidia caused by ectopic expression of the *Drosophila* receptor (Fig. 4C–E, compare to Fig. 1C). Next, we tested the ability of loss of function alleles of 48 candidate genes to modify the human ALK-induced eye phenotype. Many alleles identified in the initial modifier screen also affected the activity of all human ALK variants tested (Fig. 4F), while dose-reduction of *ave, cnk, exex*, *foxo*, *jeb*, *Mask*, *MESR3, Nhe2*, *Pi3K21B*, *Ret*, *spen*, *Taf12L*, and *Thor* had no effect. Interestingly, *P{XP}*^*d00622*^ and *Df(3L)Exel6135* suppressed the phenotype induced by both *hALK* + *ALKAL1* and *hALK-F1174L* and since neither *P{XP}*^*d00622*^ nor *Df(3L)Exel6135* had previously been connected to the function of a particular gene, we decided to analyze these genomic aberrations in more detail.

### Misexpression alleles of the DLK/LZK homolog wallenda (wnd) suppress the Alk-induced eye phenotype

**Figure 5:**
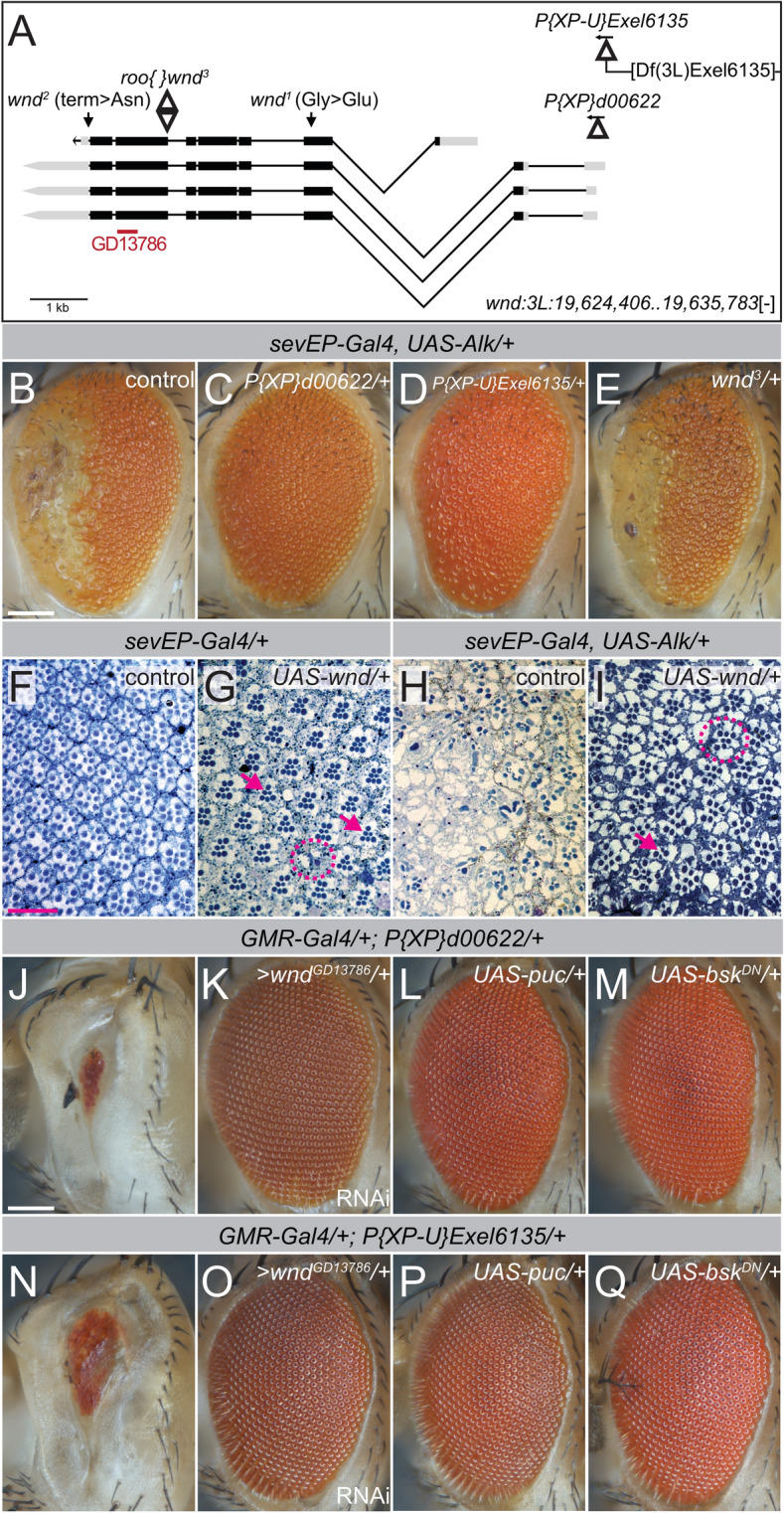
Inducible *wnd* gain-of-function alleles antagonize the effects of ectopic Alk expression. (**A**) Schematic representation of the *wnd* gene structure according to FlyBase (version FB2019_05). Black boxes represent protein-coding exons, grey boxes UTRs; lines indicate intronic regions of the four predicted *wnd*-transcripts. The positions of characterized point mutations and P-element insertions are highlighted. *P{XP}d00622* and *P{XP-U}Exel6135* (*wnd*^*d00622*^ and *wnd*^*Exel6135*^) contain *UAS*-elements (direction indicated by arrows) and are inserted in or immediately upstream of the *wnd* 5′UTR. *P{XP-U}Exel6135* is a hybrid P-element flanking the chromosomal deficiency *Df(3L)Exel6135*. Scale bar = 1 kb. (**B**–**E, J**–**Q**) Eyes of adult female flies with the indicated genotypes are shown. n > 100 animals, 100% penetrance. Scale bar is 100 µm. (**F**–**I**) To visualize ommatidia patterns in adult female *Drosophila* eyes, eye sections were stained with toluidine blue. (**F**,**G**) *sevEP-Gal4/* + *and sevEP-Gal4* > *Wnd/* + *controls.* (**H**) Visible necrotic scars and absence of ommatidia in the anterior part of an eye upon *sevEP-Gal4/* + induced *UAS-Alk/* + expression. (**I**) *Overexpression of Wnd/* + rescues ommatidia patterns in *sevEP* > *UAS-Alk flies* (H). Scale bar = 20 µm.

*P{XP}*^*d00622*^ and *Df(3L)Exel6135* were identified while mapping the suppressor activity within two partially overlapping deficiencies (*Df(3L)ED228* and *Df(3L)ED229,* Table S1) that also contained the strong suppressor candidate *Taf6*^56^. *P{XP}*^*d00622*^ is a P-element^57^ inserted in the 5´UTR region of *wallenda* (*wnd*), the homolog of human *LZK* and *DLK*^58^ (Fig. 5A,C; control shown in 5B), while *Df(3L)Exel6135* is a small deficiency with a proximal breakpoint mapped 48 bp upstream of *wnd* (Fig. 5A,D). Surprisingly, three previously characterized *wnd* loss of function alleles^58^ were unable to suppress the *sevEP-Gal4* > *UAS-Alk/* + eye phenotype (Figs. 5A,E, S3C,D), prompting us to reevaluate the *P{XP}*^*d00622*^ and *Df(3L)Exel6135* suppressors. We noted that the *P{XP}*^*d00622*^ insertion contains *UAS-*sequences that allow Gal4-driven miss-expression of nearby genes^57^. *UAS-*sequences are also present on the *Df(3L)Exel6135* chromosome that harbors a hybrid *P{XP-U}Exel6135*-element immediately upstream of the *wnd* transcription start site^57^. We therefore reasoned that the *sevEP-Gal4* driver in our screen could have induced ectopic expression of Wnd in eye discs of *sevEP-Gal4* > *UAS-Alk/*+ *; P{XP}*^*d00622*^*/*+ and *sevEP-Gal4* > *UAS-Alk/*+ *; P{XP-U}Exel6135/*+ animals*.* To test the possibility that Wnd overexpression was suppressing the Alk-induced eye phenotype, we expressed an independent *UAS-wnd* transgene^58^ in *sevEP-Gal4/*+ control and *sevEP-Gal4* > *UAS-Alk/*+ eye discs (Fig. 5F-I, Fig. S3E, F). While expression of Wnd alone (Figs. 5G, S4B) only had a subtle effect on eye morphology leading to occasionally reduced rhabdomere numbers per ommatidia (Fig. 5G, circle) and more frequent absence of single photoreceptor cells (Fig. 5G, arrows), it rescued the *sevEP-Gal4* > *UAS-Alk/* + phenotype (Figs. 5H,I, S4B). However, fused ommatidia (Fig. 5I, circle) and ommatidia with a reduced number of photoreceptor cells (Fig. 5I, arrow) could still be observed in the eye discs of these animals (Fig. 5I). Notably, *UAS-wnd* transgene expression was also able to rescue the rough-eye phenotype observed in all three activating conditions of human ALK, likely reflecting higher levels of expression of the *UAS-wnd* transgene compared to *P{XP}*^*d00622*^ and *Df(3L)Exel6135* (Table S1). Wnd has been described as a cell death regulator in the JNK pathway, and over-expression of *UAS-wnd* with *GMR-Gal4* results in cell death and a characteristic “small-eye” phenotype^33^. In line with these findings, we observed a “small-eye” phenotype when we combined *GMR-Gal4* with either *P{XP}*^*d00622*^ or *P{XP-U}Exel6135* (Fig. 5J,N, controls shown in Fig. S3). This “small-eye” phenotype was suppressed by w*nd*-specific RNAi supporting that both *P{XP}*^*d00622*^ or *P{XP-U}Exel6135* result in ectopic expression of Wnd when combined with a *Gal4*-driver (Fig. 5K,O). In agreement with the proposed role of Wnd in the JNK-pathway, co-expression of either a dominant-negative variant of the Jun kinase Basket (*UAS-bsk*^*DN*^) or the JNK-antagonist protein phosphatase Puckered (*UAS-puc*) suppressed the “small-eye” phenotype (Fig. 5L,M,P,Q). Taken together, these experiments suggest that *P{XP}*^*d00622*^ and *P{XP-U}Exel6135* are inducible gain of function (gof) alleles of *wnd* and are henceforth referred to as *wnd*^*d00622*^ and *wnd*^*Exel6135*^.

### JNK pathway activation suppresses the effect of ectopic Alk signaling

**Figure 6:**
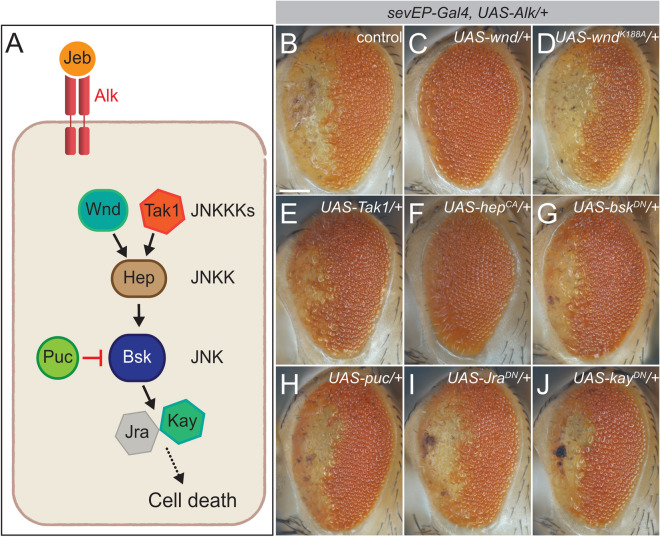
Ectopic JNK pathway activation rescues the *sevEP-Gal4* > *UAS-Alk* eye phenotype. (**A**) Schematic representation of the JNK signaling pathway and the suggested role for Alk signaling in repressing Wnd-mediated apoptosis. (**B**–**J**) Eyes of adult female flies ectopically expressing *UAS-Alk* and JNK pathway components as indicated under the control of the *sevEP-Gal4* driver. n > 50, 100% penetrance. Scale bar = 100 µm.

We next investigated the role of JNK signaling in our *sevEP-Gal4* > *UAS-Alk* model. First, we tested whether Wnd kinase activity was required to suppress the *sevEP-Gal4* > *UAS-Alk* phenotype. Expression of a kinase-dead Wnd variant (*UAS-wnd*^*K185A*^)^58^ did not rescue the rough eye phenotype, indicating that indeed Wnd kinase activity is important for the suppression (Figs. 6C,D,S4). We then addressed the effect of modulating individual components of the JNK signaling cascade on the *sevEP-Gal4* > *UAS-Alk/* + eye phenotype (Fig. 6A,B). While co-expression of the JNKKK Tak1 had a weak rescuing effect, expression of a constitutively activated variant of the JNKK Hemipterous (*hep; UAS-hep*^*CA*^) strongly suppressed the *sevEP-Gal4* > *UAS-Alk/* + eye phenotype (Figs. 6E,F, S4), suggesting that JNK activity is required for suppression. In agreement with this, blocking JNK signaling by expressing either dominant negative *basket* (*UAS-bsk*^*DN*^), *Jura (UAS-Jra*^*DN*^*)*, or *kayak* (*UAS-kay*^*DN*^) in *sevEP-Gal4* expressing cells failed to suppress the Alk-driven eye phenotype (Fig. 6G,I,J; controls in Fig. S4), as did expression of *puckered* (*UAS-puc*) (Fig. 6H), a negative regulator of JNK.

### Alk expression induces non-cell autonomous apoptosis

**Figure 7:**
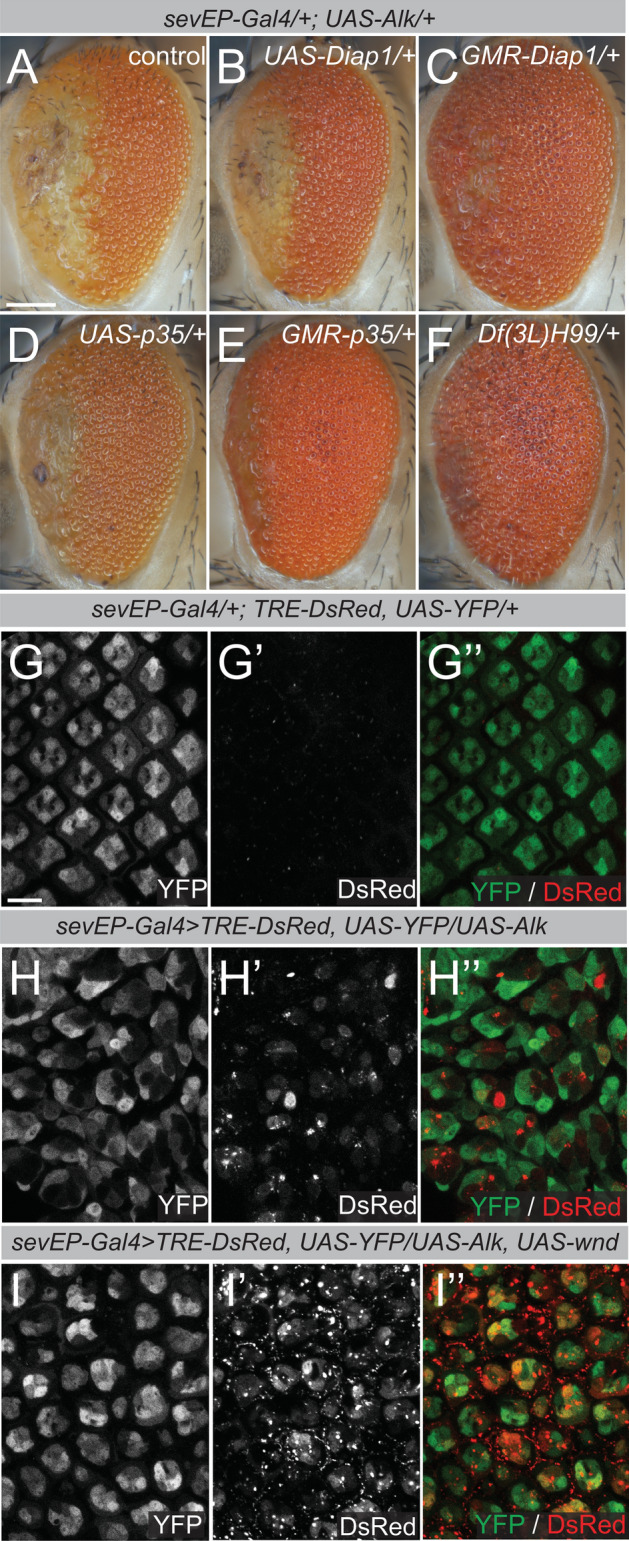
Ectopic *wnd* expression attenuates Alk-induced, JNK-mediated cell competition. (**A**–**E**) Eyes of adult female flies expressing the indicated transgene(s) under the control of the binary *Gal/UAS-* system or the *GMR* promotor. n > 75, 100% penetrance. (**F**) Phenotypic rescue of a *sevEP-Gal4* > *UAS-Alk* fly eye by one copy of *Df(3L)H99*, a deficiency for the pro-apoptotic genes *hid*, *grim*, and *rpr*. n > 40, 100% penetrance. Scale bar = 100 µm. (**G**–**I**’’) Eye discs from pupae (50 apf) expressing *sevEP-*driven *UAS-YFP* and the JNK activity reporter *TRE-RFP*. TRE-activity is not observed in controls (**G**-**G’’**) but upon ectopic Alk expression (**H**–**H**’’). (**I**–**I**’’) TRE-activity in *sevEP-Gal4* > *UAS-Alk, UAS-wnd/* + eye discs co-expressing Wnd and Alk.

Our previous experiments indicated that accumulation of Alk expressing cells in the anterior part of the fly eye correlated with the loss of ommatidia organization (Fig. 1). Therefore, it could be possible that Alk-negative neighboring cells play an important role in restricting the expansion of Alk-positive cells, eventually suppressing the phenotype in the posterior region of the eye. Supporting this assumption, broad expression of the cell death inhibitors Diap1 and p35 under direct control of the *GMR* promoter but not under *sevEP-Gal4* control, strongly suppressed the Alk-induced eye phenotype (Fig. 7A–E, controls in Fig. S5). Moreover, reducing the dose of *hid*, *grim* and *rpr* using the deficiency *Df(3L)H99* resulted in strong suppression of the Alk-induced eye phenotype (Fig. 7F), further suggesting that protecting Alk-negative neighbors from cell death enhances their ability to resist the Alk expressing cells.

**Figure 8:**
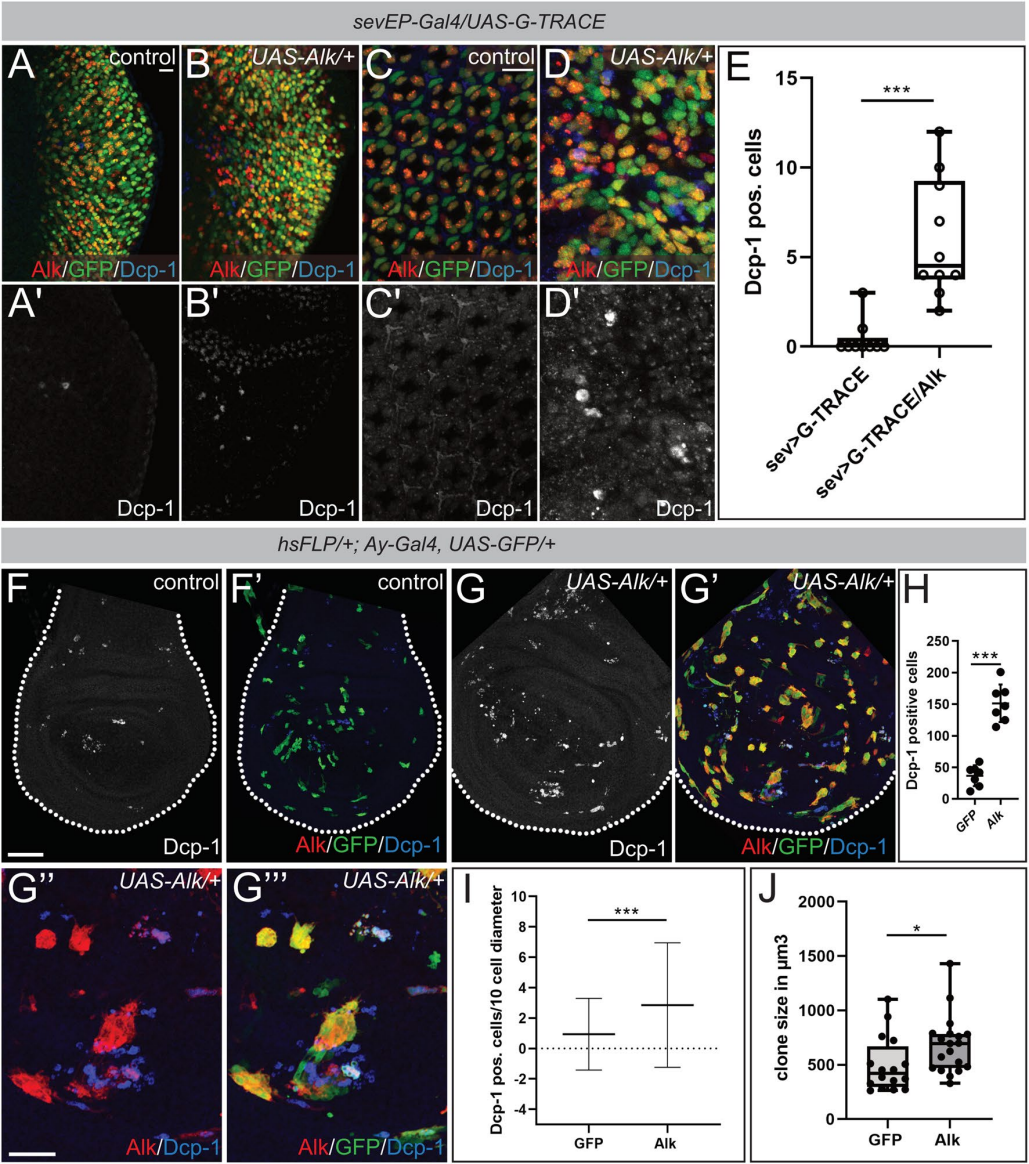
Alk-expressing cells exhibit features of super-competitors. (**A**–**D’**) Anti cleaved *Drosophila* Death caspase-1 (Dcp-1) staining of sevEP-Gal4 > UAS-G-TRACE (**A**,**A**’,**C**,**C**’) and sevEP-Gal4 > UAS-G-TRACE/UAS-Alk (**B**,**B**’,**D**,**D**’) eye discs at the wandering 3rd instar larval stage and 50 h apf. Scale bars = 10 µm. (**E**) Quantification of Dcp-1 positive cells using a bee-swarm boxplot representation. Mann Whitney Test reveals statistical significance, ***p < 0.001 (control n = 10, *UAS-Alk* n = 9). (**F**–**G’’’**) Immunofluorescence staining of Alk-expressing clones in wing imaginal discs dissected from wandering 3rd instar larvae. Discs were stained for Dcp-1, GFP and Alk. Scale bar = 50 µm in F. Scale bar = 10 µm in G’’. (**H**) Quantification of Dcp-1 positive cells in GFP control and Alk overexpressing wing discs. Bars depict means. Error bars represent S.D.; ***p < 0.001 (n = 7 wing discs). An unpaired, two-tailed t-test was applied to reveal significant differences. (**I**) Quantification of Dcp-1 positive cells in an 8–10 cell diameter surrounding GFP-control (n = 110) and Alk-overexpressing (n = 113) clones in the L3 wing discs. Means and SD (error bars) are depicted. Mann Whitney test was applied to reveal significant differences, ***p < 0.001. (**J**) Bee-swarm boxplot representation of the quantification of clone size of age-matched GFP-control (n = 16) and Alk-overexpressing (n = 19) clones in L3 wing discs. Mann Whitney test was applied to reveal significant differences, *p < 0.05.

To analyze this further, we used the *TRE-JNK* activity reporter system^59^ in *sevEP-Gal4* > *UAS-Alk/* + eye discs. No *TRE-JNK* activity was observed in control 50 h hpf eye discs expressing *UAS-YFP* under the control of the *sevEP-Gal4* driver (Fig. 7G–G’’). In Alk-expressing discs however, the *TRE-DsRed* reporter was activated in both YFP-negative and YFP-positive cells (Fig. 7H–H’’). Co-expression of Wnd in *sevEP-Gal4* > *UAS-Alk, UAS-wnd/* + eye discs resulted in strong *TRE-DsRed* reporter activity in Alk-expressing cells (Fig. 7I–I’’). One explanation for these observations could be that *sevEP-Gal4* > *UAS-Alk* expressing cells acquire a competitive advantage that is abrogated by Wnd-induced JNK activity. The role of JNK in cell competition involves both pro- and anti-tumorigenic responses and depends on the duration of JNK activation as well as on the genetic context^60,61^. For instance, JNK activation has been observed in invasive and migrating cells in *Drosophila* tumor models^62,63^, but also efficiently triggers death of cells with reduced fitness during cell competition^64–66^. To discriminate between these effects we analyzed cell death in *sevEP-Gal4* > *UAS-Alk* expressing eye discs with antibodies against cleaved Dcp-1, an effector caspase downstream of Dronc^67^. Increased Dcp-1 staining could already be observed in third instar larval eye discs, when compared to the controls (Fig. 8A–B’). G-TRACE analysis further suggested that many Dcp-1 positive cells were in close vicinity to, but not derived from, the *sevEP-Gal4* lineage (Fig. 8B,D). Increased Dcp-1 staining could still be observed in *sevEP-Gal4* > *UAS-Alk* (Fig. 8D,D’) but not in control (Fig. 8C–C’) eye discs at pupal stages (50 h apf; quantified in Fig. 8E), suggesting that Alk-expressing cells induce cell death in neighboring cells from early stages of ectopic Alk expression. To further test the hypothesis that ectopic Alk-expression provides a competitive advantage at the expense of cell death in neighboring cells we used the *hsFLP*/*Ay-Gal4* system^68^ to induce Alk-expressing clones in developing wing discs. Wing discs with Alk-expressing clones displayed significantly increased numbers of Dcp-1-labelled cells when compared to control discs with only GFP-expressing clones (Fig. 8F–G’’’, quantified in 8H). In addition, the number of Dcp-1-positive cells within a range of 8–10 cells around Alk-expressing clones was also significantly increased compared to GFP-clone controls (Fig. 8I). Moreover, Alk-expressing clones were slightly increased in size relative to GFP controls, which further supports a competitive advantage of Alk-expressing cells (Fig. 8J). Taken together, these results show that expression of Alk results in cells with enhanced fitness and suggest a role for Alk in non-cell autonomous induction of cell death in surrounding cells.

## Discussion

Malignant transformation by ALK involves several mechanisms including hyper-activation of Ras/MAPK-, PI3K- and STAT3 signaling pathways as well as increased expression of transcriptional regulators and stem-cell factors, which shifts the affected cell to a more undifferentiated state^69–72^. In this study, we conducted a genome-wide deficiency screen in *Drosophila* to identify modifiers of Alk signaling, resulting in the identification of many known and anticipated signaling molecules, such as Ras/Raf/ERK network components (MESR3, Cnk, Aveugle), PI3K21B, STAT92E, and the protein serine/threonine phosphatase Alphabet which is in agreement with previous screening efforts^73^. Amongst the signaling molecules we also found *split ends* (*spen*)^74^ and *Star*^75,76^, consistent with their known roles in RTK signaling^73^. Another gene identified as a suppressor of the Alk-induced eye phenotype was *moleskin*, which is a homologue of the vertebrate Importin-7, and has been proposed to be important for the nuclear import of activated ERK^77^. Among the identified transcription factors, Foxo and the orphan nuclear receptor transcription factor Tailless (Tll) were modifiers of Alk signaling activity. Nuclear localization of Foxo is regulated by Alk-driven AKT/PI3K signaling in insulin-producing cells in the *Drosophila* brain^23^ and, similarly, oncogenic ALK variants affect FOXO3a localization in human anaplastic large-cell lymphoma (ALCL) and neuroblastoma cell culture models^71,78^. In addition to its function in terminal patterning in the *Drosophila* embryo^52,79^, *tll* is expressed in the optic lobe primordium^80^, where it controls cell-fate decisions during Bolwig’s organ and optic lobe precursor development^81^. Interestingly, the mammalian Tll homolog TLX/NR2E1 acts as self-renewal factor in neural stem cells and neuroblastoma tumor spheres, and elevated expression of *TLX/NR2E1* is correlated with an unfavorable prognosis in neuroblastoma patients^82,83^. We also identified tissue-specific transcription factors, like the PAX6 homolog Eyeless and Eyes absent (EYA) which have been linked to human neuroblastoma^84,85^. Moreover, eight factors involved in initiation of transcription from RNA polymerase II regulated promoters (*TFIIA-S*, *TFIIA-L*, *TFIIEα*, *TFIIEβ* and the TFIID-associated factors *Taf4* (*Taf*_*II*_*110*), *Taf6* (*Taf*_*II*_*60*), *TFIIS* (*TCEA1*), and *Taf12L* (*Taf*_*II*_*30α-2)*), were revealed as downstream effectors of Alk signaling. However, mutations in core TFII-complex members could lead to a general reduction of transcriptional activation that would also affect *Alk* transgene expression. Similarly, mutations of the *AF4/FMR2* family (*AFF*) homolog *lilliputian* (*lilli*) were strong suppressors of the Alk-induced eye phenotype^86,87^. As for the identified TFII-complex members, suppression by *lilli* alleles should be interpreted with caution as they affect *hsp70*- and *sE-*promotor-dependent transgene expression and are therefore frequently found as suppressors in genetic overexpression screens^87^.

Most strikingly, inducible gain of function alleles of the MAP3K Wallenda (Wnd) that were serendipitously identified during the candidate mapping process were amongst the strongest suppressors. Wnd is one of several JNKKKs in *Drosophila* and has previously been shown to induce JNK-mediated cell death^33,34,88^. Testing other components of the JNK signaling pathway, we noted that only activators of the pathway, such as the Wnd JNKKK and an activated version of the Hep JNKK, were able to suppress the Alk-induced eye phenotype, while transgenes that inhibit JNK signaling had no effect, suggesting that activation of the JNK pathway leads to suppression of the phenotype. Interestingly, Wnd was more effective than Tak1 in suppressing the Alk-induced eye phenotype, which may reflect earlier observations that the JNKKKs provide context-dependent output, in contrast to Hep, which is part of the core JNK signaling module^33,89^. These observations led us to hypothesize that Wnd overexpression in our *sevEP-Gal4* > *UAS-Alk* model might have shifted the cells into a state where competitive advantages gained by ectopic Alk signaling are abrogated upon high levels of JNK pathway activity. A possible mechanism how Alk-expressing cells acquire this advantage could be the repression of pro-apoptotic factors like Hid upon increased Ras/ERK and PI3K/Akt signaling which not only involves direct phosphorylation of Hid by MAPK/ERK but also transcriptional regulation by Foxo and E2F1 which were also found as modifiers in our screen^90^. Alk-expressing cells activate multiple signaling pathways that result in promotion of the pro-tumorigenic response, and we know from previous work that Alk overexpression leads to activation of MAPK signaling in both eye discs and clones of imaginal discs^26^.

Cell death is a central mechanism in the process of cell competition and the JNK signaling pathway is thought to play an important role^64,91^. Work from many investigators has identified numerous context-dependent effects of JNK signaling in this process, including scenarios where cells with higher fitness (“winners”) eliminate their less-competitive neighbors by activating the JNK-pathway in these “loser-cells”^34,35,65,66,91^. Conversely, JNK activation can lead to increased cellular fitness if the apoptotic pathway downstream of JNK is blocked^60,92^. A number of genes with oncogenic potential have been reported to confer a competitive advantage on *Drosophila* cells. One of the best studied examples is Myc that transforms cells into super-competitors at the expense of surrounding cells that are induced to undergo apoptosis^66,93^, which has been reported to be mediated by both JNK-dependent^66^ and JNK-independent mechanisms^93^. A number of observations support a role for Alk in conveying a competitive advantage. Firstly, inhibiting cell death in all cells of the eye disc substantially rescued eye morphology, while blocking cell death in Alk-expressing cells alone had no effect. Secondly, expression of Alk led to an increased TRE-DsRed signal in eye discs. Thirdly, Alk-negative neighbors appear to be sensitized to undergo cell death as reflected in the increased Dcp-1 staining of wing discs containing Alk-expressing clones. Intriguingly, apoptosis is increased in the *Drosophila* eye when EGFR-triggered cell differentiation is blocked^94,95^, suggesting that undifferentiated cells involved in cell competition may be more sensitive to JNK-pathway activation, and we noted more Dcp-1 staining in the third instar larval eye disc in the region that is closest to the morphogenetic furrow which may reflect this. Finally, clones expressing Alk were slightly, but significantly increased in size compared with controls. We also noted TRE-activity in Alk-expressing cells of the *sevEP* lineage as well as YFP-negative cells which could resemble different contexts of JNK activation in the two cell populations. In that case, it would be plausible to speculate that Alk signaling could protect cells by activating the Ras/Raf/ERK pathway which in turn could inhibit pro-apoptotic effector genes. Repression of apoptosis by EGFR signaling has indeed been observed in the developing eye disc and revealed the pro-apoptotic gene *hid* as direct target of the Ras/MAPK pathway^96–98^. In addition, suppression of apoptosis by activated ALK in a ALK-F1174L/MYCN-driven zebrafish neuroblastoma model^99^, and pro-apoptotic responses in human cell cultures after ALK inhibitor treatment^100,101^ have been reported further strengthening a connection between Alk-signaling and cell-intrinsic inhibition of apoptosis.

These findings shed light on a previously unappreciated potential of Alk to induce cell death in the surrounding environment that may ultimately promote growth of Alk-expressing cells. Whether this is also true in human ALK-driven cancer remains to be elucidated, although our initial analysis with ALK-F1174L and the NPM1::ALK fusion oncogenes that are known oncogenic variants in human cancer would suggest that this may be the case. In mammalian cells, ALK signaling leads to activation of multiple signaling pathways, including anti-apoptotic responses^55^ that may act in concert to convey a competitive advantage. Moreover, as mentioned above, *Drosophila* Myc transforms cells into super-competitors^66^, which may be relevant in human cancers such as neuroblastoma where ALK is known to cooperate with MYCN to promote tumorigenesis^11,55^. In conclusion, while our screening efforts identified many genes that will require further analysis to define their role in Alk signaling, it also allowed us to identify a role for Alk in cell fitness and competition that may be of relevance in human ALK-driven cancer.

## Methods

### Drosophila melanogaster stocks and genetics

Standard *Drosophila* husbandry procedures were followed^102^. Stocks were maintained on a potato-mash-based diet at room temperature and crosses were performed in constant climate chambers at controlled 25 °C and 60% humidity levels, 12:12 h day and night cycle. Fly stocks were obtained from the BDSC (NIH P40OD018537), the KYOTO Stock Center (DGRC) at Kyoto Institute of Technology and the Vienna *Drosophila* Resource Center (VDRC) at the Campus Science Support Facilities GmbH (CSF). Other lines used were: *ave*^*CC9-36B*^ ^36^, *C3G*^*MS*^ ^43^, *cnk*^*ΔY2H*^ ^36^, *jeb™*^*.3xOLLAS*^ (this study), *gzl*^*Δ2*^ ^103^, *UAS-Alk.L* ^26^, *UAS-hALKAL1.G* and *UAS-hALKAL2.G* ^4^, *UAS-hALK*^*F1174L*^ ^104^, *UAS-hNPM1::hALK* (this study), *UAS-Jra.DN, UAS-kay.DN* ^105^, *UAS-puc.M* ^106^, *tll*^*g*^ ^107^, and *wnd*^*2*^ ^58^.

### Generation of jeb™^.3xOLLAS^ flies

We used CRISPR/Cas9 genome editing to induce homology-directed repair (HDR) in the *Drosophila jeb* locus^108,109^. The *pBluescriptII KS[-]* HDR donor construct (GenScript) contained homology arms (corresponding to 357 bp immediately upstream and 406 bp downstream of the *jeb* stop codon) that flanked a central segment encoding for a six amino acid linker (Gly-Ser-Gly-Gly-Ser-Ala), the transmembrane domain of *Drosophila* Neurotactin (Ala325-His347), a triple OLLAS-tag and a TAA stop codon. Two CRISPR target sites (AGCGGATGCGTTACTACGCTGGG and TGTGTGTGCTGTGTATTAGACGG) in close proximity to the endogenous *jeb* stop codon were identified using the flyCRISPR optimal target finder tool^109^. Targeting gRNAs were cloned into *pU6-BbsI-chiRNA* (Addgene) as described^109^. Donor and *U6-chiRNA* plasmids were injected into *y*^*1*^* M{vas-Cas9}ZH-2A* embryos (BestGene Inc.). HDR-events were identified by single fly PCR screening using a primer combination specific to the knocked-in sequence (5′-GTTCCTCTTGCAGAGTGTCCT-3′ and 5′-CTAATTGTTTGTCA-ATGTGAGTTCGCT-3′). Candidates were further analyzed by Sanger sequencing (GATC Services—Eurofins Genomics) and tested for non-complementation with the *jeb*^*weli*^ null allele^18,36^.

### Construction of the UAS-hNPM1::hALK transgene

We employed the deduced amino acid sequence of the human nucleophosmin 1 (hNPM1)-human anaplastic lymphoma kinase (hALK) chimera reported by Morris et al. (^8^, GenBank: AAA58698.1) as template to generate the *UAS-hNPM1::hALK* transgene. gBlock fragments (Integrated DNA Technologies) of the hNPM1 n-terminus and the hALK kinase domain were assembled into EcoRI/XbaI-cut *pUASTattB* vector using the NEBuilder HiFi assembly cloning kit (NEB). The construct was verified by Sanger sequencing (GATC Services—Eurofins Genomics) and injected in *y*^*1*^* w*^*67c23*^*; P{y*^+*t7.7*^ = *CaryP}attP1* embryos (BestGene Inc.).

### Genetic screening

Transgenic flies ectopically expressing the Alk receptor in a subset of cells of the developing ommatidia (*sevEP-Gal4, UAS-Alk/CyO*) exhibit a temperature-sensitive phenotype. Stocks reared at 29 °C exhibited pupal lethality while a characteristic eye phenotype (Fig. 1C) is observed at 25 °C. Screening was performed under controlled conditions (constant 25 °C and 60% humidity levels, 12:12 h day and night cycle). *sevEP-Gal4, UAS-Alk/CyO* flies were crossed to stocks of the DrosDel deficiency collection kit^32^ which offers the advantage of an isogenic background and high-percentage coverage of the *Drosophila* genome. More than 20 F1 female flies per cross that did not exhibit balancer chromosome-associated markers were scored for modifications of the eye phenotype. Suppression was defined as reduction or absence of the area of “undifferentiated cells” in the anterior part of the eye. Expansion of this area, as well as dark spots and excessive necrotic tissue were scored as “other modifications” (detailed in Table S1). All scored flies had to exhibit a similar effect on the *sevEP-Gal4, UAS-Alk* phenotype although slight variations in the phenotypic strength of the observed effect were tolerated. Crosses that did not yield viable offspring of the desired genotype were performed in triplicate before assigning them to the “lethal/no progeny” group. Non-curly winged female offspring of *sevEP-Gal4, UAS-Alk/CyO* and the DrosDel project starter stock (#5905) were used as controls. Deficiencies that modified the eye phenotype in this initial screen were tested again to ensure that the results were reproducible. In order to map down the various modifier regions, we scored additional stocks carrying either smaller chromosomal deficiencies, or available mutations in loci located within the suppressing region, or *Gal4/UAS*-based transgenic constructs of the associated genes. Alleles used to reveal the modifying effect of the identified candidates are listed in Table S1. More than 20 non-balanced F1 female flies per cross were scored, crosses with each allele were performed at least twice. We did not score for partial suppression/modification but slight variations in phenotypic strength were tolerated. For the human ALK modifier experiment, more than 15 non-balanced F1 female flies per cross were scored for modifications of the induced “rough-eye” phenotype. Only complete penetrance was scored, and crosses were performed twice.

### Antibody staining and clonal induction

Dissection and antibody staining of imaginal discs was carried out according to^110^. Alk- or GFP-expressing clones were generated with *hsFLP*/*Ay-Gal4* system^68^. Briefly, freshly hatched first larvae were picked at 24–26 h after egg laying (AEL), prior to 30 min heat shock at 54–56 h AEL to induce overexpression clones in imaginal discs. Animals were dissected at wandering third instar larval stage (104–106 AEL) and analyzed. The following antibodies were purchased from the Developmental Studies Hybridoma Bank (DSHB, created by the NICHD of the NIH and maintained at The University of Iowa, Department of Biology, Iowa City, IA 52242) and used in the specified dilutions: mouse anti-Cut (CT; 2B10; 1:500), mouse anti-Prospero (PROS; MR1A; 1:100), rat anti-*Drosophila* epithelial cadherin (DECad; DCAD2; 1:100), rat anti-embryonic lethal abnormal vision (Elav; 7E8A10; 1:500). Other antibodies used: guinea pig anti-*Drosophila* Alk (Alk, 1:1,000, ^2^), rabbit anti β-Galactosidase pre-absorbed on *w*^*1118*^ embryos (β-Gal, Cappel #0855976, 1:1,500), rabbit anti-cleaved *Drosophila* Death caspase-1 (Dcp-1; Asp216; 1:200, Cell Signaling #9578), chicken anti-GFP (1:500; Abcam ab13970), rabbit anti-RFP (1:1,000; Abcam ab62341). Secondary antibodies and animal sera were purchased from Jackson ImmunoResearch. Stained samples were embedded in Fluoromount-G (SouthernBiotech, #0100-01) and analyzed with a ZEISS Axio Imager.Z2 microscope. Images were acquired with a ZEISS LSM800 confocal microscope using the ZEN Blue edition software.

### Preparation of adult Drosophila eyes for thin sectioning and microscopic analysis

To visualize ommatidia patterns, heads from adult flies were dissected and fixed in a mix of 2% glutaraldehyde, and 4% formaldehyde in 50 mM sodium cacodylate buffer (pH 7.4) and post-fixed in 4% Osmium and 2% KFeCN in 200 mM sodium cacodylate buffer (pH 7.4) followed by stepwise dehydration in 30%, 50%, 70%, 85%, 95%, 100% ethanol. Samples were embedded in Hard Plus Resin 812 (Electron Microscopy Sciences) for 48 h at 60 °C. Eye Section (1 µm) were obtained using a Reichert-Jung Ultracut E ultramicrotome with Ultra 45^0^ diamond knife (Electron Microscopy Sciences) on glass slides and stained with a 1% toluidine-blue solution. Images were obtained using a ZEISS Axio Imager.Z2 microscope equipped with a ZEISS Axiocam 503 color camera and the ZEN Blue edition software.

### Imaging of adult Drosophila eyes

Image stacks of adult fly eyes were acquired with a ZEISS AxioZoom.V16 stereo zoom microscope equipped with LED ring light and an Axiocam 503 color camera. The enhanced depth of focus (EDF) module of the ZEN Blue edition software was used for further image processing.

### Databases, data analysis and statistics

Genomic coordinates refer to the Dmel_Release_6 sequence assembly. Detailed information about *Drosophila* stocks and genetics is available on FlyBase (https://flybase.org/), the Database of *Drosophila* Genes & Genomes (Thurmond et al., 2019). Modifying loci (as listed in Table S1) were analyzed by PANTHER14.0-driven gene ontology (GO) enrichment analysis (https://geneontology.org/)^51^). Interaction analysis of Alk modifying loci (Fig. 3B, listed in Table S1) was analyzed with STRING version 11 (https://string-db.org)^111^ employing a medium confidence score of 0.4 for interactions. GraphPad Prism 7 software was used for all statistical analyses.

## Supplementary information

Supplementary Figure 1.

Supplementary Figure 2.

Supplementary Figure 3.

Supplementary Figure 4.

Supplementary Figure 5.

Supplementary Table 1.

Supplementary legends
