# Supplementary figures and images for: Identification of the Wallenda JNKKK as an Alk suppressor reveals increased competitiveness of Alk-expressing cells
Source: Sci Rep. 2020 Sep 11;10:14954. doi: 10.1038/s41598-020-70890-6 (PMC7486895; doi:10.1038/s41598-020-70890-6)

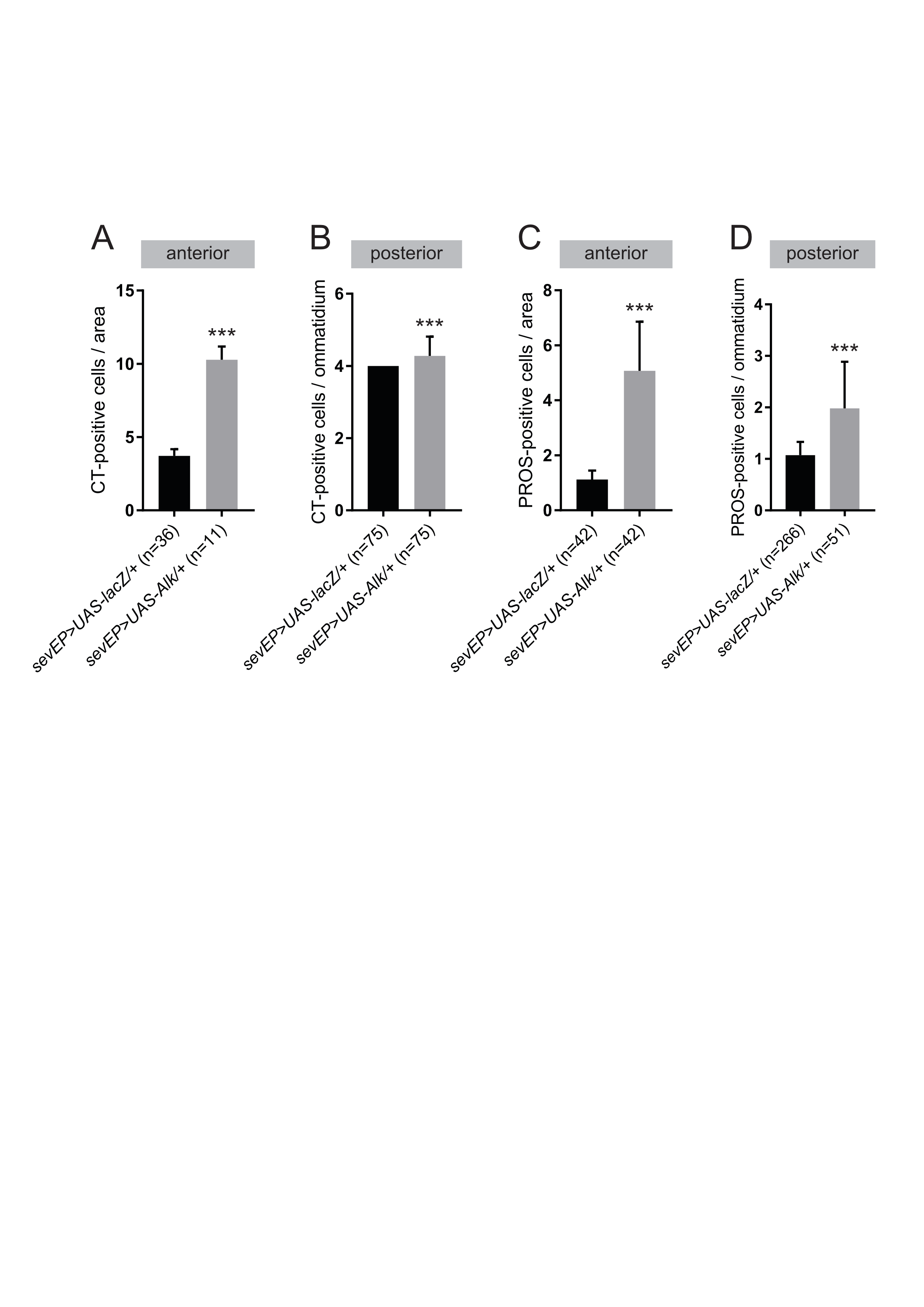

Supplement: Supplementary file 1 — Supplementary Figure 1. [file 41598_2020_70890_MOESM1_ESM.tiff]

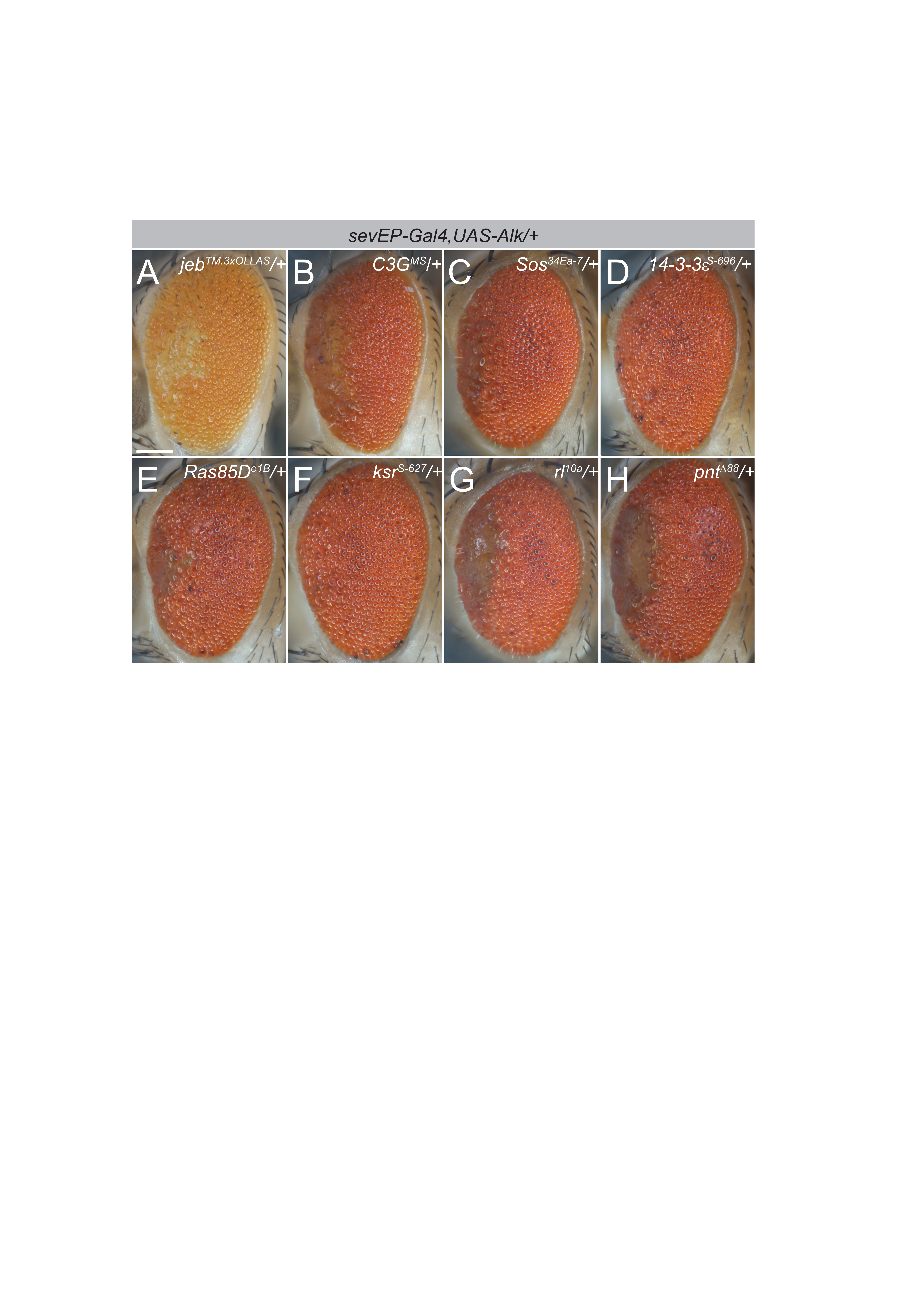

Supplement: Supplementary file 2 — Supplementary Figure 2. [file 41598_2020_70890_MOESM2_ESM.tiff]

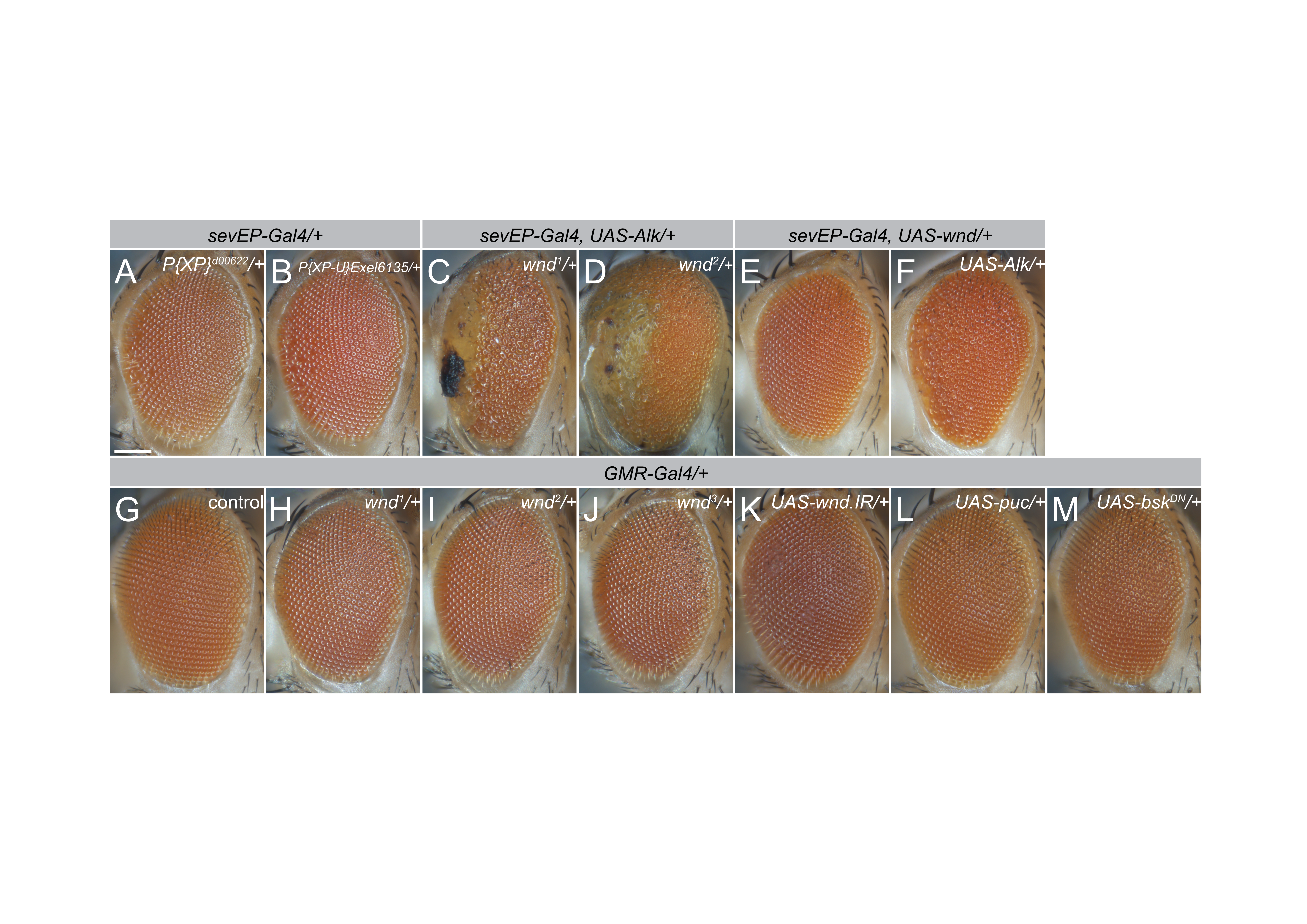

Supplement: Supplementary file 3 — Supplementary Figure 3. [file 41598_2020_70890_MOESM3_ESM.tiff]

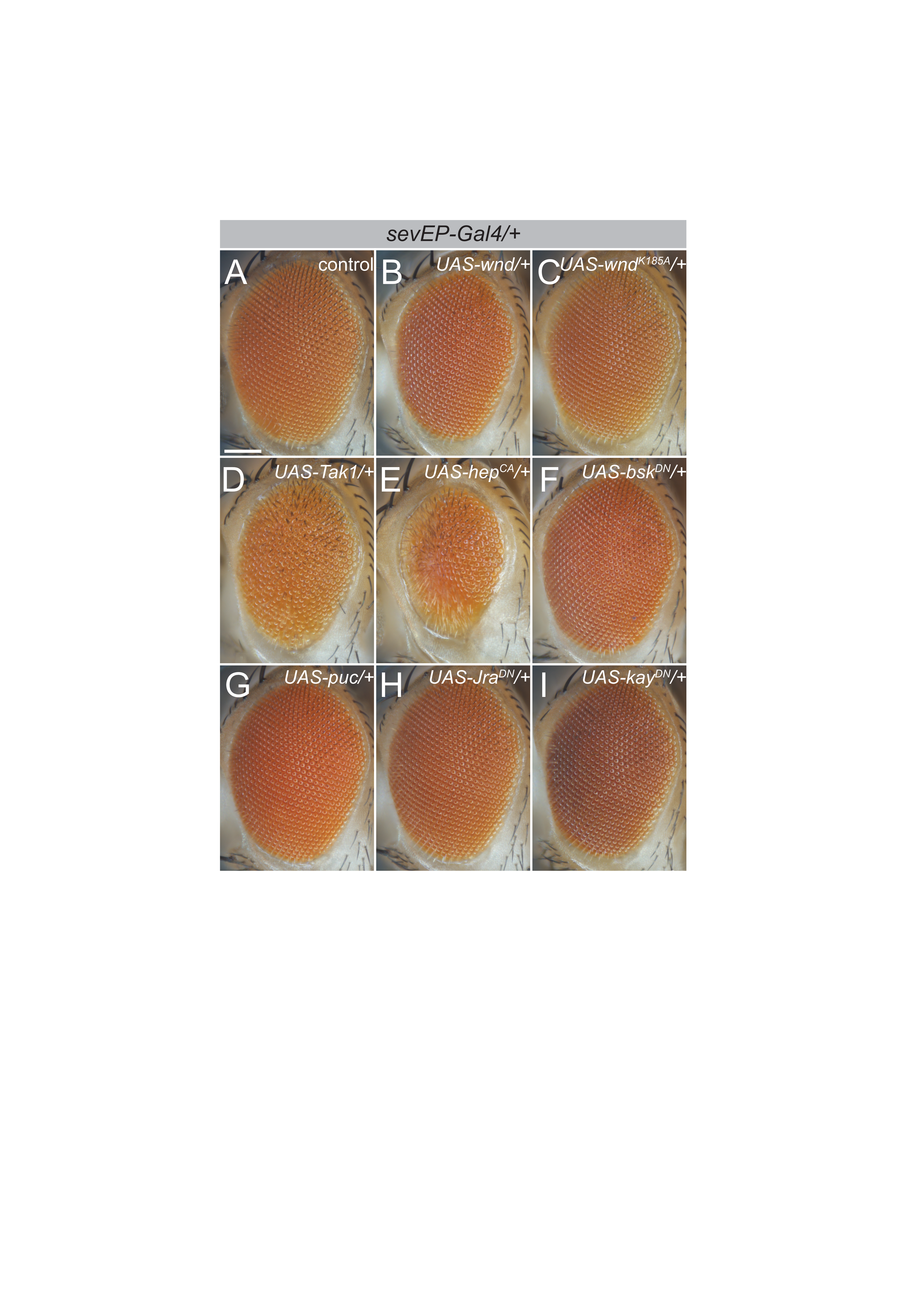

Supplement: Supplementary file 4 — Supplementary Figure 4. [file 41598_2020_70890_MOESM4_ESM.tiff]

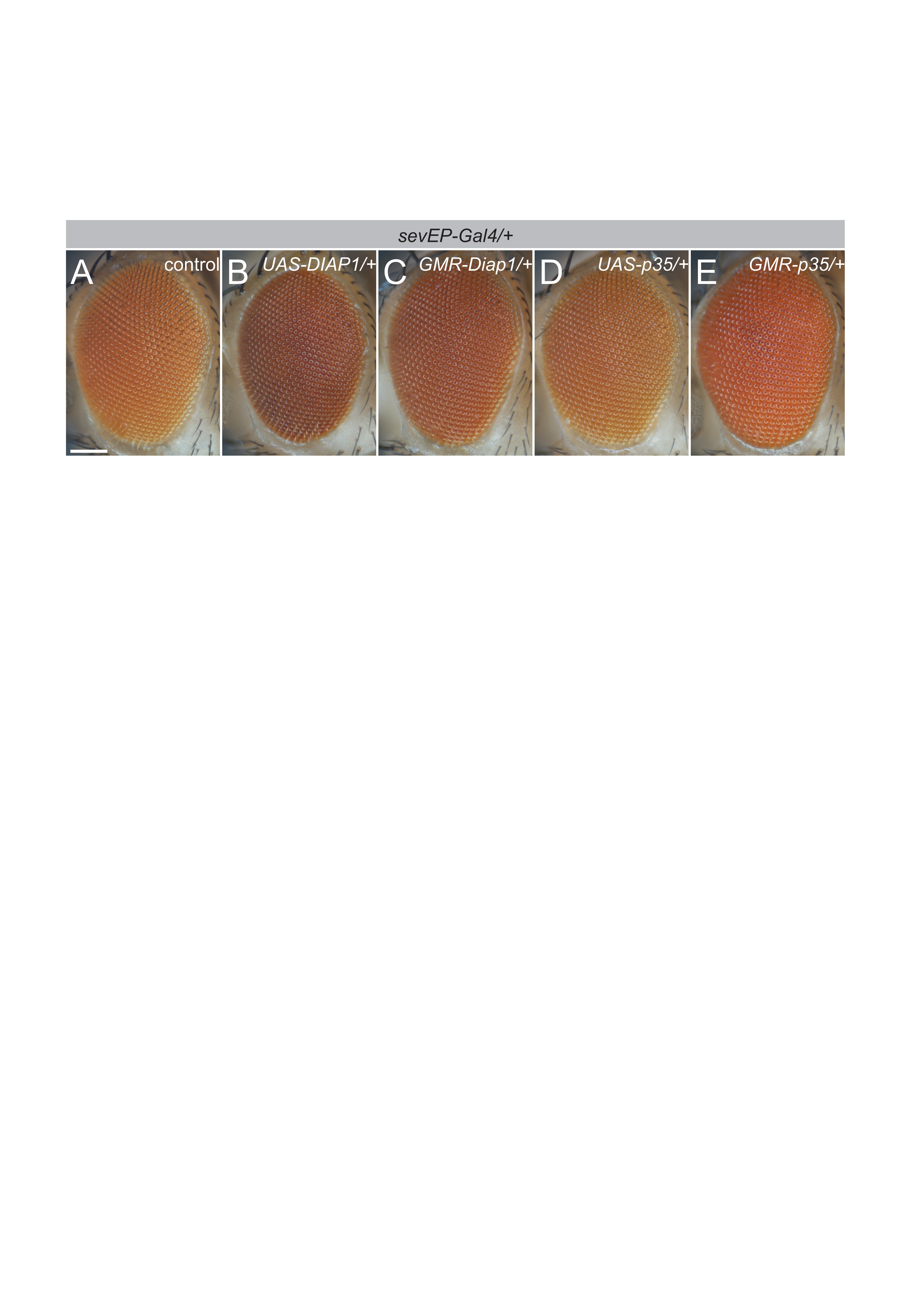

Supplement: Supplementary file 5 — Supplementary Figure 5. [file 41598_2020_70890_MOESM5_ESM.tiff]
