# Supplementary material for: Identification of the Wallenda JNKKK as an Alk suppressor reveals increased competitiveness of Alk-expressing cells
Source: Sci Rep. 2020 Sep 11;10:14954. doi: 10.1038/s41598-020-70890-6 (PMC7486895; doi:10.1038/s41598-020-70890-6)
Supplement: Supplementary file 7 — Supplementary legends [file 41598_2020_70890_MOESM7_ESM.docx]

**Supplementary figures**

**Supplementary Figure S1**

Ectopic expression of *UAS-Alk* with the *sevEP-Gal4* driver interferes with normal eye development. Columns depict means of either CT-positive **(A, B)** or Pros-positive **(C, D)** cells counted per area (10 µm x 10 µm) in the prospective anterior part of the developing eye (A, C) or per ommatidium in the posterior part (B, D). Error bars represent S.D.; ***p < 0.001. Mann-Whitney *U*-test was applied to reveal significant differences. (A) Mean of CT-positive cells per area in *sevEP-Gal4/+* (6.94) vs. *sevEP-Gal4, UAS-Alk/+* (20.3); n ≥ 11 areas. (B) CT-positive cells in *sevEP-Gal4/+* ommatidia (4) vs. mean (4.28) of CT-positive cells in *sevEP-Gal4, UAS-Alk/+* ommatidia; n = 75 ommatidia. (C) Mean of Pros-positive cells per area in *sevEP-Gal4/+* (1.12) vs. *sevEP-Gal4, UAS-Alk/+* (5.07); n = 42 areas. (D) Mean of Pros-positive cells in *sevEP-Gal4/+* ommatidia (1.07) vs. *sevEP-Gal4, UAS-Alk/+* (1.98); n ≥ 51 ommatidia.

**Supplementary Figure S2**

Suppression of the *sevEP-Gal4>UAS-Alk/+* induced eye phenotype by selected candidates. Eyes of adult female flies carrying one copy of the loss of function alleles *jeb^TM.3xOLLAS^* **(A)**, *C3G^MS^*  **(B)**, *Sos^34Ea-7^* **(C)**, *14-3-3e^S-696^* **(D)**, *Ras85D^e1B^* **(E)**, *ksr^S-627^* **(F)**, *rl^10a^* **(G)** or *pnt^Δ88^* **(H)** are shown. Scale bar = 100 µm; anterior is left.

**Supplementary Figure S3**

Controls Fig. 5: Inducible *wnd* gain-of-function alleles antagonize the effects of ectopic Alk expression. Eyes of adult female flies are shown. **(A, B)** *P{XP}d00622* and *P{XP-U}Exel6135* under control of the *sevEP-Gal4/+* driver. **(C, D)** *wnd^1^* and *wnd^2^* do not suppress the *sevEP>UAS-Alk/+* induced eye phenotype. **(E, F)** UAS-*wnd* suppresses the *sevEP>UAS-Alk/+* induced eye phenotype. **(G-M)** Wild-type eye morphology of *GMR-Gal4/+* controls (G), and *GMR-Gal4/+* flies heterozygous for different *wnd* alleles as indicated (H-J), co-expressing *wnd-*specific RNAi (*wnd.IR* in K), *UAS-puc* (L) or *UAS-bsk^DN^* (M). Scale bar = 100 µm; anterior is left.

**Supplementary Figure S4**

Controls Fig. 6: Ectopic JNK pathway activation rescues the *sevEP-Gal4>UAS-Alk* eye phenotype. Eyes of adult female *sevEP-Gal4/+* control flies **(A)** and flies expressing *UAS-*transgenes encoding for JNK pathway components as indicated with the *sevEP-Gal4/+* driver **(B-I)** are shown. Scale bar = 100 µm; anterior is left.

**Supplementary Figure S5**

Controls Fig. 8: Ectopic *wnd* expression attenuates Alk-induced, JNK-mediated cell competition. Eyes of adult female *sevEP-Gal4/+* fly **(A)**, and flies expressing *UAS-Diap1* under *sevEP-Gal4/+* control **(B)**, or *GMR-Diap1* **(C)** which is expressed independently of the *Gal4/UAS* system are shown. **(D)** Fly expressing *UAS-p35* under *sevEP-Gal4/+* control. **(E)** Fly expressing *GMR-p35* independently of *sevEP-Gal4/+*. Scale bar = 100 µm; anterior is left.

**Supplementary Table S1 including 4 Worksheets:** (1) DrosDel Alk modifier screen, (2) Identified candidates, (3) PANTHER14.0-driven GO-enrichment analysis, (4) Human Alk modifiers.
